# Trappin-2/Elafin Modulate Innate Immune Responses of Human Endometrial Epithelial Cells to PolyI∶C

**DOI:** 10.1371/journal.pone.0035866

**Published:** 2012-04-24

**Authors:** Anna G. Drannik, Kakon Nag, Xiao-Dan Yao, Bethany M. Henrick, Jean-Michel Sallenave, Kenneth L. Rosenthal

**Affiliations:** 1 Department of Pathology & Molecular Medicine, McMaster Immunology Research Centre, Michael G. DeGroote Institute for Infectious Disease Research, McMaster University, Hamilton, Ontario, Canada; 2 Unité de Défense Innée et Inflammation, Institut Pasteur, Paris, France; 3 Unité U874 INSERM, Paris, France; 4 Université Paris 7-Denis Diderot, Paris, France; University of Cape Town, South Africa

## Abstract

**Background:**

Upon viral recognition, innate and adaptive antiviral immune responses are initiated by genital epithelial cells (ECs) to eradicate or contain viral infection. Such responses, however, are often accompanied by inflammation that contributes to acquisition and progression of sexually transmitted infections (STIs). Hence, interventions/factors enhancing antiviral protection while reducing inflammation may prove beneficial in controlling the spread of STIs. Serine antiprotease trappin-2 (Tr) and its cleaved form, elafin (E), are alarm antimicrobials secreted by multiple cells, including genital epithelia.

**Methodology and Principal Findings:**

We investigated whether and how each Tr and E (Tr/E) contribute to antiviral defenses against a synthetic mimic of viral dsRNA, polyinosine-polycytidylic acid (polyI∶C) and vesicular stomatitis virus. We show that delivery of a replication-deficient adenovector expressing Tr gene (Ad/Tr) to human endometrial epithelial cells, HEC-1A, resulted in secretion of functional Tr, whereas both Tr/E were detected in response to polyI∶C. Moreover, Tr/E were found to significantly reduce viral replication by either acting directly on virus or through enhancing polyI∶C-driven antiviral protection. The latter was associated with reduced levels of pro-inflammatory factors IL-8, IL-6, TNFα, lowered expression of RIG-I, MDA5 and attenuated NF-κB activation. Interestingly, enhanced polyI∶C-driven antiviral protection of HEC-Ad/Tr cells was partially mediated through IRF3 activation, but not associated with higher induction of IFNβ, suggesting multiple antiviral mechanisms of Tr/E and the involvement of alternative factors or pathways.

**Conclusions and Significance:**

This is the first evidence of both Tr/E altering viral binding/entry, innate recognition and mounting of antiviral and inflammatory responses in genital ECs that could have significant implications for homeostasis of the female genital tract.

## Introduction

Genital epithelial cells (ECs) provide the first line of defense against sexually-transmitted infections (STIs) [1], [2]. Upon viral sensing through pattern-recognition receptors (PRRs), ECs initiate innate and adaptive immune responses that serve to eradicate or contain viral pathogens [3], [4]. ECs can directly respond to viruses and viral pathogen-associated molecular patterns (PAMPS) by secreting innate protective factors, including defensins and cathelicidins [5] as well as members of the whey-acidic protein (WAP) family [6]. Of the 18 human WAP proteins, only a few have been well characterized to date [7], and among the better understood are serine antiproteases elafin (E) with its precursor, trappin-2 (Tr), as well as secretory leukocyte protease inhibitor (SLPI), and prostate stromal protein 20 kDa (ps20) [7], [8].

The physiological role of serine antiproteases has been extensively studied over the past two decades [9], mainly due to their contribution to homeostatic equilibrium through the control of proteases, inflammation, and infections [10], [11]. Together with other proteins, such as snake venom neurotoxins [12] and whey acidic protein [13], serine antiproteases share an evolutionary conserved canonical cysteine-rich four-disulfide core (FDC) domain, or the WAP domain, involved in protease inhibition [14]. Trappin-2 (9.9 kDa) (or pre-elafin) is a secreted and unglycosylated protein of 95-amino acids (aa) [15] that contains an N-terminal cementoin domain (38-aa) [16] and elafin (5.9 kDa), a C-terminal inhibitory WAP (57-aa) domain [14], [16]. Elafin is released from the N-terminus of Tr by proteolysis, arguably most efficiently by mast cell tryptase [17], [18]. Antiprotease activity and wound repair were the first described properties of Tr and E (Tr/E), similar to SLPI. Unlike ps20, SLPI along with Tr/E are functional neutrophil serine protease inhibitors [7], [11]. Inhibition of human neutrophil elastase (HNE) and proteinase 3 by the inhibitory loop on a WAP domain allows Tr/E to control tissues proteolysis associated with excessive inflammation in a neutrophil-rich environment. In turn, cross-linking between repeated hexapeptide motifs (GQDPVK) on the N-terminal portion of each Tr/E [15], [19] and extracellular matrix proteins arguably allows Tr/E to repair compromised tissue integrity [19], [20]. In addition, due to their cationic nature, but not exclusively [21], Tr/E were shown to possess antimicrobial activity against Gram-negative and Gram-positive bacteria [21], [22], [23] and certain fungal infections [24]. Worth mentioning is that similar to SLPI, antibacterial activity of Tr/E appeared to be independent of their antiprotease function [22]. Later, anti-inflammatory features of the antiproteases were also described, showing that Tr/E and SLPI were capable of reducing activation of NF-κB and AP-1 by altering IκB activation [25] and proteosomal degradation [26], respectively, in response to inflammatory and bacterial stimulation. More recent studies, however, also reported immunomodulatory properties of Tr/E. Indeed, depending on the environment, Tr/E can either dampen inflammation [20], [26] or promote immunostimulatory events and prime the immune system [27], [28]. Both Tr/E are found at mucosal surfaces [6], [29], in tissues [30], [31], [32], [33], [34] and multiple cell types, including genital ECs [6], [31] and regarded as alarm antiproteases, as they are mainly produced in response to pro-inflammatory stimuli like LPS [35], TNFα [36], and IL-1β [31], [37]. Interestingly, ECs from the female genital tract (FGT) produce Tr/E constitutively, with uterine cells capable of producing even greater amounts of Tr/E in response to a viral ligand, polyinosine-polycytidylic acid (polyI∶C) [6], indicating the significance of these molecules in controlling the local milieu in the FGT.

Viral double-stranded RNA (dsRNA) is a PAMP generated during the life cycle of most, if not all, viruses [4], [38]. Double-stranded RNA, including viral dsRNA and its synthetic mimic polyI∶C, are recognized by at least two families of PRRs: Toll-like receptors (TLRs), including TLR 3 [39], [40], and RNA helicases, namely retinoic acid inducible gene-I (RIG-I) [41], [42] and melanoma differentiation associated gene 5 (MDA5) [43]. Following recognition of dsRNA, activated PRRs initiate a series of signaling events, triggering phosphorylation, homodimerization and translocation into the nucleus of a set of transcription factors like interferon (IFN) regulatory factor 3 (IRF3), IRF7, NF-κB, and ATF2/c-Jun (c-Jun) [38], [41], [44], [45]. Inside the nucleus, these transcription factors either work independently or interact with each other [46] in triggering the transcription of antiviral and inflammatory gene products, such as type I IFNs and IL-8, IL-6, and TNFα. Specifically, IRF3 alone can directly bind to the IFN-stimulated response element in the promoter region of interferon-stimulated genes (ISGs) and activate a set of ISGs and their products in the absence of type I IFN production [47], [48]. Such antiviral cascade is devoid of excessive inflammatory responses and is induced when a low viral stimulation is detected. Alternatively, in response to a high viral load, IRF3 associates with NF-κB and c-Jun to trigger the production of IFNβ and the induction of ISGs in an IFN-dependent mode [45], [49], which also triggers robust inflammation, target cell recruitment, and/or tissue damage due to the production of pro-inflammatory cytokines and chemokines along with antiviral type I IFNs molecules [39], [48].

Treatment with polyI∶C has been shown to induce potent antiviral protection *in vitro* and *in vivo*, making polyI∶C an attractive candidate for microbicide or vaccine adjuvant trials against STIs [39], [40], [50]. However, in addition to antiviral activity, polyI∶C also triggers the release of pro-inflammatory mediators [39], [40], therefore potentially negating its beneficial effects in the FGT. While pro-inflammatory factors are important for immune cell recruitment and activation, if poorly controlled, they may also be detrimental in FGT, since acquisition and pathogenesis of common STIs are associated with immune activation and inflammation [51], [52], [53]. Hence, interventions leading to better control of inflammatory responses may prove to be more beneficial for increased antiviral protection and overall health in the FGT. Recently, new evidence has accumulated on the role of Tr/E in protection against viruses [6], [29]. Indeed, Tr/E have been associated with resistance to HIV mucosal transmission in commercial sex workers (CSWs) in Kenya [29]. Later, Ghosh *et al.* reported an anti-HIV feature of E in *in vitro* study [6]. Furthermore, Roghanian *et al.* documented that Tr expression increased Ad/LacZ viral clearance, as well as secondary immune responses, in a murine model *in vivo* [27].

Collectively, these data clearly demonstrate the importance of Tr/E in antiviral protection, although specifically how Tr/E contribute to antiviral immune-inflammatory responses is still unknown. Thus, the objective of this study was to elucidate whether and how Tr/E contribute to innate antiviral and inflammatory responses in uterine ECs elicited by a viral ligand, polyI∶C. Here, we describe novel antiviral and immunomodulatory properties of Tr/E. To the best of our knowledge, this is the first study to present evidence of Tr/E affecting host innate recognition and modulating antiviral and inflammatory responses in genital ECs against polyI∶C.

## Materials and Methods

### Reagents

PolyI∶C and lipopolysaccharide (LPS) were reconstituted in the phosphate-buffered saline (PBS) and used at concentrations shown in figures (Sigma-Aldrich, Oakville, ON, Canada). Human recombinant (r) proteins Tr (rTr) (R&D Systems, Burlington, ON, Canada) [15] and in-house recombinant E (rE) (described below) were used in *in vitro* experiments and as reference markers for Western blotting. The amount of Tr/E being used in this study ranges from 0.2 to 5 µg/ml to cover the physiological levels (within 1 µg/ml) reported previously [6], [29] as well as concentrations achieved in supernatants of human endometrial carcinoma (HEC)-1A cells infected with a replication-deficient adenovirus (Ad) expressing human Tr gene (Ad/Tr) (over 1 µg/ml).

### Cell lines

HEC-1A, Caco-2 (human colonic epithelial cells), and A549 (derived from a type II human alveolar cell carcinoma) cells [54] were obtained from American Type Culture Collection (Rockville, MD). HEC-1A and A549 cells were cultured in McCoy's 5A Medium Modified (Invitrogen Life Technologies, Burlington, Ontario, Canada) and DMEM, respectively, supplemented with 10% fetal bovine serum (FBS), 1% HEPES, 1% l-glutamine (Invitrogen Life Technologies), and 1% penicillin-streptomycin (Sigma-Aldrich) at 37°C in 5% CO_2_. Caco-2 cells were cultured in DMEM growth medium containing 5% FBS, 1% l-glutamine, 1% penicillin-streptomycin, 5 mL NEAA, and 9.6 mL NaHCO_3_.

### Adenoviral constructs and delivery in cell culture

The Ad constructs used in this study have been described in detail elsewhere [55], [56], [57]. To express human Tr, the Ad/Tr vector, encoding gene for 95-aa human Tr, was used [55], [56]. This Ad construct was previously called Ad/E. E1, E3-deleted empty adenovirus Ad-dl703 (Ad/dl), coding for no transgene, was used as a control for Ad/Tr [57]. Both Ad vectors were prepared at the Centre for Gene Therapeutics at McMaster University (Hamilton, ON, Canada). To generate supernatants containing Tr, HEC-1A cells were infected with MOI 0–50 plaque-forming units (PFU) of Ad/Tr (Ad/Tr-cells) or Ad/dl (Ad/dl-cells) overnight at 37°C in Opti-MEM® I Reduced Serum Medium (Invitrogen Life Technologies), washed with PBS and incubated for 12 h in serum-containing medium. Cells were washed again and incubated for additional 24 h in serum-free cell culture medium. Cell-free supernatants were used either for protein measurement by ELISA or for antiprotease activity against HNE. Another aliquot of supernatants was further concentrated with 3–30 kDa MWCO centrifugal filter units (Amicon, Millipore, Billerica, MA, USA) as per supplier's instructions and used in *in vitro* studies. For routine experiments, epithelial cells were either treated with Opti-MEM medium alone (UT) or with MOI 50 PFU of Ad/dl or Ad/Tr at 37°C overnight. After PBS washes and rest for 4 h, cells were incubated in serum-containing medium alone or with polyI∶C for additional 24 h.

### Vesicular stomatitis virus (VSV) plaque reduction assay and antiviral assay

Vesicular stomatitis virus (VSV-GFP), a lytic IFN-sensitive virus expressing green fluorescent protein under the viral promoter (a kind gift from Dr. Brian Lichty, McMaster University), was used in a plaque reduction assay [44] to assess the role of Tr/E in antiviral protection. This method is based on determining the ability of VSV-GFP to replicate in cell cultures in presence of biologically active antiviral factors, e.g., IFNs [44]. Briefly, HEC-1A cells were seeded in 96-well culture plates and infected with Ad (Ad-cells) as described above, followed by treatment with medium alone or polyI∶C for 24 h. Induction of antiviral response was assessed by subsequently challenging cell monolayers in serum-free medium with MOI 1 PFU of VSV-GFP. GFP fluorescence intensity was visualized 24 h later on a Typhoon Trio (Amersham Bioscience, GE Healthcare Bio-Sciences Corp., Piscataway, NJ, USA) and quantified using Image Quant 5.2 software.

### MTT viability assay

MTT assay, described elsewhere [39], was used as per supplier's instructions to determine viability of Ad-exposed and polyI∶C-treated HEC-1A cells (Biotium Inc., Hayward, CA, USA).

### ELISA assays

Cell-free supernatants were stored at −70°C until assayed for human Tr/E, IL-8, TNFα, IL-6 with ELISA Duoset kit (R&D Systems), human IFNβ by ELISA kit from Antigenic America Inc. (Huntington Station, NY, USA), and IFNα subtypes by *VeriKine™* Human Interferon-Alpha Multi-Subtype ELISA Kit (Piscataway, NJ, USA) according to the supplier's protocol. Analytes were quantified based on standard curves obtained using an ELISA reader Tecan Safire ELISA reader (MTX Labs Systems Inc.). Cut off limit for Tr/E and IL-8 was 31.25 pg/ml; for TNFα and IFNβ was 15.6 pg/ml; for IFNα subtypes was 12.5 pg/ml, and levels detected below these limits were considered as undetectable.

### Generation of recombinant human elafin

To prepare rE protein, cDNA fragments encoding the relevant part of Tr (amino acid residues A61–Q117) were amplified from HEC-1A cells cDNA using the following primers: 5′-ACAGGATCCGCGCAAGAGCCAGTCAAAGGTCCA-3′ and: 5′-CAGGAATTCTCACTGGGGAACGAAACAGGCCATC-3′. The amplified elafin-cDNA was gel purified, restriction digested, and directionally subcloned into *Bam*H I and *Eco*R I site of bacteria expression vector pHAT10 (BD Biosciences, Rockville, MD, USA) in frame to the HAT-tag. The clone was confirmed by restriction digestion and nucleic acid sequencing of both strands. The plasmid was transformed into Escherichia coli BL21 strain (Codon Plus, Palo Alto, CA, USA). When the cells grew in Luria-Bertani broth containing 100 µg/ml ampicillin to an A_600_ of 0.55–0.60 at 37°C, protein expression was induced by adding isopropyl-β-D-thiogalactopyranoside (IPTG) to a final concentration of 1 mM for 6 h. The cells were harvested by centrifugation at 2,000×*g* for 5 min, and cell pellets were washed once with ice-cold PBS (pH 7.4) and resuspended in wash buffer (50 mM Na_2_PO_4_, 300 mM NaCl, pH 7.0). The desired recombinant protein, rE, was found in the soluble fraction in pilot experiment. After disrupting the cells by freeze-thaw and subsequently by sonication, the lysates were centrifuged at 20,000×*g* for 20 min, and the soluble fraction was collected. The soluble recombinant protein was purified from this fraction by BD TALON metal affinity resin (BD Biosciences, Mississauga, ON, Canada) according to the manufacturer's instructions and dialyzed against PBS at 4°C. The expressed human rE was confirmed by Western blotting targeting the HAT-tag as well as by specific antibody for human E.

### Preparation of cell extracts and Western blot (WB) analysis

Whole-cell extracts were prepared by using whole-cell extract buffer (50 mM Tris-HCl, pH 8.0, 150 mM NaCl, 1%NP-40, 1%SDS, 1×protease inhibitor (Roche, Mississauga, ON, Canada)) as per standard protocol. Protein amount was quantified using Bradford assay with bovine serum albumin (BSA) (Sigma-Aldrich) as a standard and Bio-Rad Dye Reagent Concentrate as a protein stain (Bio-Rad Laboratories, Mississauga, ON, Canada). WB was performed on a 10% polyacrylamide denaturing SDS-PAGE gel and PVDF membranes (Amersham, Arlington Heights, IL, USA) as per standard protocol, using the following primary antibodies: anti-human Tr/E TRAB2O (Hycult Biotech, Uden, Netherlands), tIRF3 FL-425 (Santa Cruz Biotechnology, Santa Cruz, CA, USA), pIRF3 Ser396 (Cell Signaling, Danvers, MA, USA), RIG-I #4520 (Cell Signaling), MDA-5 R470 (Cell Signaling), c-Jun (60A8) (Cell Signaling), p-c-Jun (Ser73) (Cell Signaling), TLR3 IMG-5631-2 (Imgenex, San Diego, CA, USA), NF-κB p65 (C-20): sc-372 (Santa Cruz), GAPDH ab9485 (Abcam) antibodies at a dilution of 1∶1000, except for GAPDH (1∶5000) and p-NF-κB p65 (Ser 536): sc-33020 (Santa Cruz) (1∶100). After incubation with corresponding horse-radish peroxidase (HRP)-conjugated secondary antibody (Bio-Rad Laboratories), blots were visualized using a SuperSignal West Femto or Pico Chemiluminescent Substrate kit (Thermo Scientific, Rockford, IL, USA). GAPDH was used as internal loading control. Quantification of band intensities was done using MBF_ImageJ for Microscopy Software.

### Transfection and luciferase assay

HEC-1A cells (5×10^5^ per well) were transfected in 0.5 mL of Opti-MEM® medium (Invitrogen Life Technologies) in a 24-well plate with 20 ng of DNA of each of pgkβ-Gal and pNF-κB-Luc or pAP-1-Luc (Stratagene, La Jolla, CA, USA) plasmids (total DNA 40 ng per well) and 50 MOI of Ad/dl or Ad/Tr overnight at 37°C utilizing Lipofectamine 2000 (Invitrogen Life Technologies) as per supplier's instructions. Pgk-β-Galactosidase plasmid was used as a normalization control. After transfection, cells were extensively washed with PBS and allowed to rest for 4 h followed by stimulation with polyI∶C and LPS for 2–4 h. After ligand stimulation, cells were washed twice with cold PBS, lysed with 1× reporter buffer (Enhanced Luciferase Assay Kit (Cat # 556866, BD Pharmingen) and subjected to one freeze-thaw cycle, after which cell lysates were collected and assayed in a 96-well plate for luciferase (Enhanced Luciferase Assay Kit, BD Pharmingen) and β-galactosidase (Luminescent β-Gal assay, Promega, Madison, WI, USA) activities separately as per supplier's protocol, using Opticomp I luminometer (MGM instruments).

### IRF3 knockdown by RNA interference

Small interfering (siRNA) molecule (Invitrogen Life Technologies) targeting IRF3 (GenBank accession number NM_001571) within 247–1530 ORF through the following sequences: IRF3-498 (start) CAACCGCAAAGAAGGGTTGCGTTTA, or non-targeting siRNA, RNAi Negative Control (medium GC content), 12935-300, (Invitrogen Life Technologies) were used to specifically knockdown IRF3. Transfections of siRNA (8 pmol) were done using Lipofectamine RNAiMAX and Opti-MEM® I Reduced Serum Medium (Invitrogen Life Technologies) as per supplier's instructions. HEC-1A cells, 3×10^4^ in a 100 µl of total volume of complete growth medium, were transfected in a 96-well BD Falcon culture plate (BD Biosciences) for 48 h before adding MOI 50 PFU of Ad/dl or Ad/Tr. Knockdown efficiency was monitored using WB.

### RNA extraction and real-time quantitative PCR analysis

The protocol for RNA isolation was described elsewhere [52]. Briefly, total RNA was isolated from Ad-cells cultured with medium alone or polyI∶C for 6 h, using TRIzol reagent (Invitrogen Life Technologies) according to the supplier's protocol. RNA was DNase-treated with DNA-free (Ambion, Austin, TX, USA) and complementary (cDNA) was synthesized from total RNA using SuperScript reverse transcriptase III (Invitrogen Life Technologies) as per supplier's protocol. Real-time quantitative PCR was performed in a total volume of 25 µl using 1× Universal PCR Master Mix (Applied Biosystems, Foster City, CA, USA), 5 µl of diluted cDNA, 500 nmol forward primer, 500 nmol reverse primer (Mobix; McMaster University, ON, Canada), and 200 nmol probe in a 96-well plate. TaqMan oligonucleotide primers and probes labeled with 6FAM at 5′-end and a non-florescent quencher at 3′-end were designed using Primer Express 1.5 (Applied Biosystems) and selected following the TaqMan rules of Applied Biosystems. The sequences were as follows: RIG-I: 5′-AGGGCTTTACAAATCCTGCTCTCTTCA-3′ (probe), 5′-GGTGTTCCAGATGCCAGAC-3′ (forward), 5′-TTCCGCAAATGTGAAGTGTATAA-3′(reverse); MDA5: 5′-TTTGGCTTGCTTCGTGGCCC-3′(probe), 5′-TGATTCCCCTTCCTCAGATAC-3′(forward), 5′-TGCATCAAGATTGGCACATAGT-3′(reverse); TLR3: 5′-TGTGGATAGCTCTCC-3′(probe), 5′-CCGAAGGGTGGCCCTTA-3′(forward), 5′-AAGTTACGAAGAGGCTGGAATGG-3′ (reverse); 18S rRNA: 5′-CGGAATTAACCAGACAAATCGCTCCA -3′ (probe), 5′-GTGCATGGCCGTTCTTAGTT-3′ (forward), 5′-TGCCAGAGTCTCGTTCGTTAT-3′ (reverse). The expression of 18S ribosomal RNA (rRNA) was used as an internal control. PCR was run with the standard program: 95°C 10 min, 40 times of cycling 95°C 15 sec and 60°C 1 min in a 96-well plate with an ABI PRIZM 7900HT Sequence Detection System using the Sequence Detector Software 2.2 (Applied Biosystems). To determine the expression of ISG56, a semi-quantitative RT-PCR was performed with oligonucleotide primer sequences (Mobix): 5″-GACAGGAAGCTGAAGGAGAAA-3′ (445-bp product) (forward), 5′-TcTTGCATTGTTTCTTCTACCACT-3′ (reverse). PCR program was as follows: 94°C for 2.5 min, 30 cycles of 94°C for 20 sec, 55°C for 30 sec, 72°C fir 1 min, 72°C for 5 min. PCR products were electrophoresed on 2% agarose gel using a 100-bp DNA ladder (Invitrogen Life Technologies) as a marker to identify PCR products. The gel was stained with a loading fluorescent dye EZ-vision N472-Q (AMRESCO Inc., Solon, OH, USA) and visualized with UV transilluminator (Gel Doc 2000, BioRad, Mississauga, ON, Canada). Densities of DNA bands were quantified to signal volumes using ImageQuant 5.0.

### Immunofluorescence staining

Immunofluorescence staining was performed as described earlier [39], [44], but with minor modifications. Ad-cells, grown on an 8-well BD Falcon culture slides (BD Biosciences), were medium- or 25 µg/ml polyI∶C-treated for 4 h, fixed with 4% paraformaldehyde, permeabilized with 0.1% Triton X-100 in PBS for 20 min, and blocked for 1 h at ambient temperature in a blocking solution (0.1% Triton X-100, containing 5% goat serum/BSA). IRF3 was detected using 1∶100 dilution in blocking solution of IBL18781 (IBL, Gunma, Japan) antibody for 1 h. Negative control rabbit immunoglobulin fraction (DakoCytomation, Glostrup, Denmark) served as an isotype control and was diluted to match the protein content of the primary anti-IRF3 antibody. Secondary antibody, Alexa Fluor 488 conjugated goat anti-rabbit IgG (Molecular Probes, Eugene, OR, USA) was added to cells in a blocking solution for 1 h. The nuclei were visualized by staining with propidium iodide. Images were acquired using an inverted laser-scanning confocal microscope (LSM 510, Zeiss, Oberkochen, Germany).

### Protease inhibition assay (PIA)

This assay was performed by measuring inhibition of HNE activity by Tr/E as was described earlier [20], but with minor modifications. Elastase-inhibitory activity was measured in a 96-well plate by combining cell-free undiluted supernatant (generated as described earlier in Materials and Methods and before or after polyI∶C treatment) (final volume 10 µl/well), serially diluted each rTr and rE, or medium alone, to a known quantity of purified HNE (50 ng in 10 µl/well) or diluent alone (negative control), and incubating for 30 min at 37°C. Subsequently, 50 µl of HNE substrate, *N*-methoxysuccinyl-Ala-Ala-Pro-Val *p*-nitroanilide (Sigma-Aldrich), diluted to 50 µg/ml in 50 mM Tris, 0.1% Triton, 0.5 M sodium chloride, pH 8 buffer were added and the hydrolysis was recorded by monitoring the increase of absorbance at 405 for 15 min using a Tecan Safire ELISA reader (MTX Labs Systems).

### Statistical analysis

Data were expressed as means ± standard deviation (SD). Statistical analysis was performed with either unpaired Student's *t* test or a one-way analysis of variance (ANOVA) using Sigma Stat 2.03.

## Results

### Ad/Tr-cells secrete Tr and both Tr/E are detected in Ad/Tr-cells supernatants in response to polyI∶C

**Figure 1:**
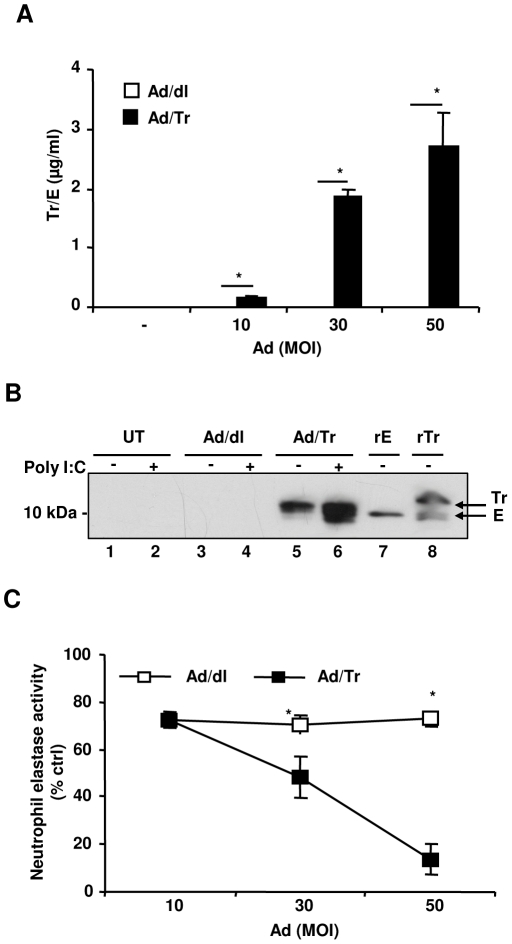
Ad/Tr-cells secrete Tr that is functionally active against HNE and both Tr/E are detected in Ad/Tr-cells supernatants in response to polyI∶C. HEC-1A cells were either treated with medium alone or with MOI 10–50 of Ad/dl or Ad/Tr. Supernatants were collected 36 h after Ad removal and tested for Tr/E expression by ELISA (*A*) or for antiprotease activity (*C*), where results are expressed as percent reduction over a positive control (media alone plus HNE and a substrate). The data are representative of two independent experiments performed in triplicate and are shown as the mean ± SD. Statistical analysis was performed using Student's *t* test with * representing significant difference between the groups, p<0.05. (*B*) Immunoblotting analysis of supernatants from HEC-1A cells developed using TRAB20 antibodies. Cells were either left untreated (lanes 1,2) or treated with MOI 50 of Ad/dl (lanes 3,4) or Ad/Tr (lanes 5,6) and then incubated for 24 h in presence of medium alone (−) or 25 µg/ml polyI∶C (+). Two recombinant reference proteins, namely in-house HAT-E (rE) (lane 7) and commercial 6×His-Tr (rTr) (lane 8), were used as comparative markers for E and Tr. Bands corresponding to the forms of Ad-induced Tr and E are indicated on the blot by arrows.

Initial studies evaluated the expression of the secreted Tr from Ad/Tr-cells by ELISA that detects both Tr/E [6]. We report that Ad/Tr-cells secreted significant amounts of Tr/E in an Ad-dose-dependent manner (Figure 1A). In contrast, untreated HEC-1A (UT) and Ad/dl-cells expressed very low to no Tr/E (Figure 1A). Since the antibodies used in the ELISA did not distinguish Tr from E in Ad/Tr-cells supernatants (Ad/Tr-sups), WB was performed to clarify the presence of both Tr/E [58]. Two recombinant reference proteins, namely commercial 6×His-Tr (rTr) [15] and in-house HAT-E (rE), were used as comparative markers for Tr and E, respectively. Due to tag insertion, both reference proteins appeared 3–4 kDa higher than the appearance of untagged proteins would be expected. Thus, rE band appeared at ∼10–11 kDa, and rTr at ∼13–15 kDa. Additionally, a smaller band ∼10–11 kDa was also observed in the rTr reference protein (Figure 1B, lane 8), being most likely E present in the protein preparation. This smaller band also appeared higher than would be expected for E, due to the His-tag insertion. Hence, we conclude that the commercial rTr is a mixture and contains both Tr/E that are indicated as such on WB by arrows (Figure 1B, lane 8). WB data demonstrate that supernatants of UT and Ad/dl-cells supernatants (Ad/dl-sups) did not show any detectable forms of endogenous Tr/E in the blot (Figure 1B, lane 1–4). In contrast, WB of Ad/Tr-sups in the absence of polyI∶C stimulation revealed protein bands that appeared between Tr/E bands of the reference proteins and thus were considered as Tr (Figure 1B, lane 5). The bands in lane 5 represent secreted Tr that resulted from Ad/Tr infection of HEC-1A cells. Further, following polyI∶C treatment of Ad/Tr-cells, the intensity of the Tr bands in Ad/Tr-sups was greatly increased, indicating a significantly higher amount of Tr (Figure 1B, lane 6) being produced. Interestingly, a smaller protein band also appeared (Figure 1B, lane 6) that was below the level of rE-reference marker protein, as well as the E band in the rTr reference protein (Figure 1B, lane 7), and thus was considered as E. Taken together, these results indicate that Ad/Tr-cells secrete Tr, and both Tr/E are detected in the supernatants following Ad/Tr-cells stimulation with polyI∶C.

### Ad/Tr-cells secrete Tr that is functionally active against HNE

Being foremost a protease inhibitor, Tr in Ad/Tr-sups was tested in protease inhibition assay [20]. The results of anti-protease assay showed that Ad/Tr-sups significantly inhibited HNE activity in a dose-dependent manner, compared to Ad/dl-sups (Figure 1C). Following polyI∶C stimulation, Ad/Tr-sups, containing presumably both Tr/E, were also tested and found functional against HNE (data not shown), comparable to before polyI∶C treatment activity.

### Tr/E significantly reduce VSV-GFP infection and enhance polyI∶C-driven antiviral protection in Ad/Tr-cells

**Figure 2:**
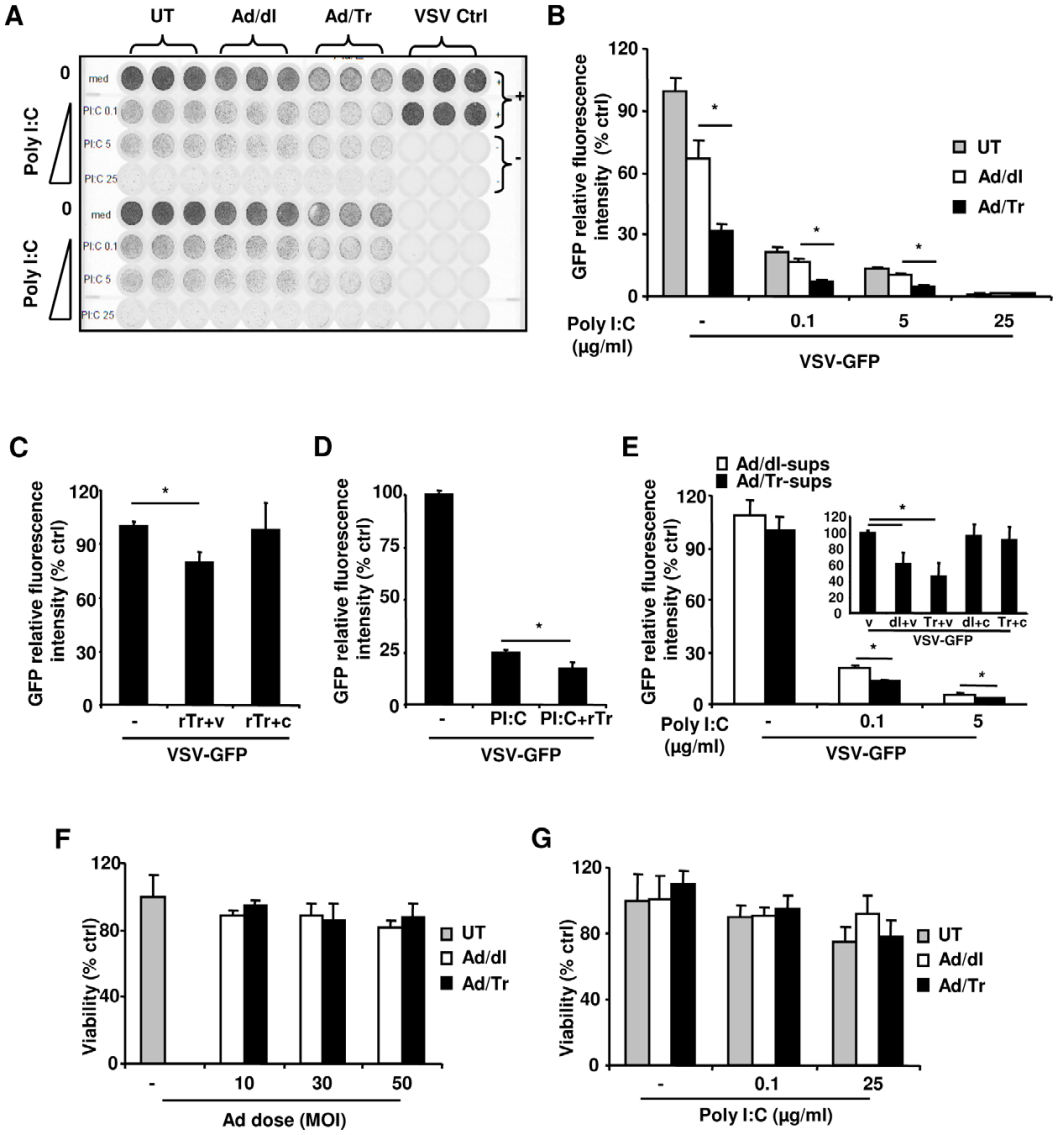
Tr/E significantly reduce VSV-GFP infection and enhance polyI∶C-driven antiviral protection in Ad/Tr-cells. Untreated (UT) or treated with MOI 50 of Ad/dl or Ad/Tr HEC-1A cells were medium- or polyI∶C 0.1–25 µg/ml-treated for 24 h, followed by MOI 1 VSV-GFP. GFP fluorescence intensity was visualized 24 h later using a Typhoon scanner (*A*), and relative fluorescence intensity to virus positive control was determined and presented as % of UT control (*B*). (*C*) VSV-GFP load after pre-incubation of 5 µg/ml of rTr either with MOI 1 of VSV-GFP (rTr+v) or cells (rTr+c) for 1 h, followed by their washing and addition of VSV-GFP. Medium-treated VSV-GFP (v) served as a positive control. (*D*) HEC-1A cells were treated with 5 µg/ml of rTr for 1 h in serum-free medium, after which medium or 0.1 µg/ml polyI∶C was added for 24 h followed by MOI 1 of VSV-GFP. This polyI∶C dose was chosen because anti-inflammatory effects of secreted/soluble proteins, compared to Ad/Tr-cells, were less potent. (*E*) HEC-1A cells were pretreated with equal volumes of supernatants from Ad/dl-cells (Ad/dl-sups) and Ad/Tr-cells (Ad/Tr-sups, 5 µg/ml final concentration) for 1 h before medium or 0.1 or 5 µg/ml polyI∶C was added for 24 h followed by MOI 1 VSV-GFP. Lower polyI∶C doses were used to better visualize VSV-GFP viral replication 24 h later, using a Typhoon scanner. Insert in panel (*E*) depicts VSV-GFP load after pre-incubation of same volumes and concentrations of Ad/dl- and Ad/Tr-sups with either MOI 1 VSV-GFP (dl+v) or (Tr+v), respectively, or cells (dl+c) or (Tr+c) for 1 h, followed by washing and addition of MOI 1 VSV-GFP. Medium-treated VSV-GFP (v) served as an untreated control. The data are representative of at least two independent experiments performed in triplicate and shown as mean ± SD. Statistical analysis was performed using ANOVA or Student's *t* test in (*C*), and * representing significant difference, when p<0.05. (*F* and *G*) Cell growth and metabolic activity of HEC-1A cells were determined by standard MTT Cell Viability Assay Kit following infection with MOI 10–50 of Ad/dl or Ad/Tr (*F*) or after stimulation with increasing doses of polyI∶C (*G*). Cell viability was expressed as % of untreated cells, which served as a negative control group, and was designated 100%; the results are expressed as % of negative control.

Since polyI∶C stimulation of ECs triggers the induction of antiviral protection [39], [44], we next determined whether Tr/E could modulate antiviral protection. Results of standard VSV-GFP plaque reduction assays show that the delivery of Ad/Tr significantly reduced VSV-GFP replication in polyI∶C-untreated cells (Figure 2A and 2B) that was beyond the anti-viral protection induced by Ad delivery alone (i.e., in Ad/dl group). Ad delivery is known to activate innate immune responses [59], and the fact that susceptibility of HEC-1A cells to VSV-GFP infection (Figure 2B) was already reduced by treating the cells with Ad/dl alone confirms the Ad-induced innate activation. Since polyI∶C-untreated Ad/Tr-cells secrete only Tr (Figure 1B), we conclude that the presence of Tr was associated with significantly increased antiviral protection of Ad/Tr-cells in the absence of polyI∶C. Following polyI∶C treatment, VSV-GFP replication was further reduced across the groups in Ad-cells (Figure 2A and 2B); viral replication in Ad/Tr-cells also remained significantly reduced (with up to 50% reduction, p<0.05) and antiviral protection increased, compared to polyI∶C-treated Ad/dl-cells (Figure 2A and 2B). Based on these results and those shown in Figure 1B, we deduce that the presence of both Tr/E was associated with significantly increased cellular antiviral protection in Ad/Tr-cells after polyI∶C treatment. Altogether, these observations clearly indicate that exogenous Tr expression in Ad/Tr-cells before VSV-GFP challenge has a separate antiviral protective mechanism in addition to the Ad- and polyI∶C-induced responses; furthermore, both Tr/E appear to mediate enhanced polyI∶C-induced cellular antiviral protection in Ad/Tr-cells.

We next attempted to elucidate specific mode(s) of Tr/E antiviral activity. Given the possibility of additional Tr/E being released from Ad/Tr-cells after polyI∶C stimulation and upon VSV-GFP challenge and thus potentially acting on both virus and cells, we tested if both or each Tr/E individually were acting through two separate mechanisms: (i) direct antiviral activity exerted during the virus/cell encounter and viral binding/entry, targeting either virus or cells; and (ii) indirect cell-associated immunomodulatory activity that targets polyI∶C-triggered cellular antiviral responses and protection. To minimize the Ad-associated effects, we used secreted/soluble proteins delivered to Ad-uninfected HEC-1A cells. Because we could not use Ad/Tr-sups after polyI∶C stimulation as a source of both Tr/E, since they would contain residual polyI∶C and thus mask the effect of Tr/E alone, a commercial rTr (with a C-terminus His-tag) was used as a source of both Tr/E, based on results shown in Figure 1B. For comparative assessment of each Tr/E individually, we used Ad/Tr-sups before polyI∶C stimulation as a source of secreted Tr (no tag), and rE (with an N-terminus HAT-tag) was used as a source of E. All the proteins were initially compared for their antiprotease activity and found equally potent against HNE at around 10 µg/ml (data not shown). Figure 2C shows that when virus, but not cells, was pre-treated with recombinant rTr for 1 h before addition onto cells, viral replication was significantly reduced by about 20% (p<0.05), compared to media alone. Of note, no further reduction in viral replication was noticed when a higher dose of rTr was used. These results clearly indicate that rTr (a mixture of Tr/E) has a statistically significant, albeit modest, antiviral effect, which appears to be due to direct, or virus-mediated, activity of the proteins. We further show that pretreatment of HEC-1A cells with rTr, followed by co-culturing with polyI∶C for 24 h before VSV-GFP challenge, significantly reduced viral replication (up to 30%, p<0.05) and enhanced polyI∶C-induced antiviral protection, compared to polyI∶C alone (Figure 2D). Interestingly, when cells were pretreated with rTr, then washed and challenged with VSV-GFP 24 h later without prior polyI∶C stimulation, no significant decrease in viral replication was observed (data not shown), suggesting that rTr does not induce potent antiviral cellular responses without polyI∶C stimulation and reduces VSV-GFP replication only following direct contact with virus. Collectively, these results suggest that secreted/soluble rTr, as a mixture of both Tr/ E, demonstrated two distinct properties: a virus-mediated antiviral activity and the modulation/enhancement of polyI∶C-induced cellular antiviral responses, suggesting that the presence of both Tr/E is required for both of these activities.

Next we assessed antiviral properties of individual secreted/soluble Tr/E preparations. We show that VSV-GFP replication was reduced (up to 40%, p<0.05) in Ad/Tr-sups-treated cells (Figure 2E), compared to controls, but only after polyI∶C treatment (Figure 2E, main graph and insert), suggesting that Tr from Ad/Tr-sups does not exhibit potent direct antiviral activity, but is capable of enhancing polyI∶C-induced cellular responses. Insert in Figure 2E demonstrates that, although a trend toward a decreased viral replication was noted when Ad/Tr-sups were pre-incubated with the virus, there was no significant inhibitory effect observed from Ad/Tr-sups, compared to their controls. Furthermore, surprisingly, in contrast to rTr or Ad/Tr-sups, rE did not exhibit any antiviral properties (data not shown), indicating differential antiviral properties of the tested proteins that could be potentially related to their structural differences. Collectively, these results clearly indicate that both Tr/E appear to be required for antiviral protection mediated via both mechanisms.

### Exogenous expression of Tr and polyI∶C stimulation do not lead to impaired cell viability or metabolic activity of Ad/Tr-cells

Considering that polyI∶C can induce apoptosis [60], we determined whether Ad/Tr-cells had impaired cell viability and metabolic activity, using a standard MTT Cell Viability Assay [39]. Our results determined no significant changes in viability and metabolic activity among the groups following either Ad, with recovery period of 4 h versus 24 h (data not shown), or polyI∶C treatment (Figure 2F and 2G). Therefore, we can exclude the impairment in the treated cells as the cause of differences between Ad/Tr- and Ad/dl-cells.

### Ad/Tr-cells respond to polyI∶C treatment with modulated production of IFNβ

**Figure 3:**
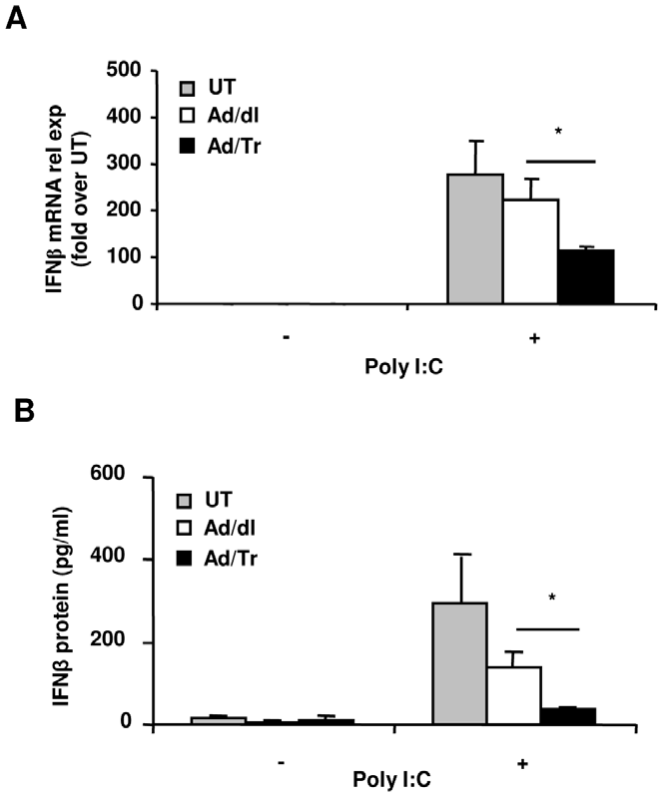
Ad/Tr-cells respond to polyI∶C treatment with reduced production of IFNβ. HEC-1A cells were either treated with medium alone (UT) or with MOI 50 of Ad/dl or Ad/Tr and incubated for 6–24 h in presence of media or 25 µg/ml of polyI∶C. (*A*) At 6 h post treatment, IFNβ mRNA from total RNA was determined by real-time quantitative RT-PCR. Values are normalized to a housekeeping gene 18S in the same sample and presented as fold induction over UT cells in absence of polyI∶C treatment. (*B*) At 24 h of polyI∶C stimulation, supernatants were tested for IFNβ expression by ELISA. Data are representative of at least two independent experiments performed in triplicate and expressed as the mean ± SD, shown in pg/ml. Statistical analysis was performed using Student's *t* test with * representing significant difference between the groups, p<0.05.

We next elucidated the immunomodulatory effect of Tr/E in Ad/Tr-cells in context of polyI∶C stimulation. Type I IFNs are considered a hallmark of antiviral response, with IFNβ being a key correlate of polyI∶C-induced antiviral protection [39], [40], [44]. We determined whether enhanced antiviral protection in presence of Tr/E was associated with higher levels of IFNβ, using quantitative real-time RT-PCR and commercial ELISA. Figure 3 demonstrates that polyI∶C treatment of Ad-cells triggered a significant induction of IFNβ expression/secretion in contrast to untreated Ad-cells. Interestingly, Ad/Tr-cells responded to polyI∶C treatment with significantly dampened, but not completely abrogated, levels of IFNβ, compared to Ad/dl-cells. Since IFNβ levels were reduced at both mRNA (Figure 3A) and protein (Figure 3B) levels, it is likely that IFNβ expression was affected primarily at the transcription level. A similar dampening effect was observed when IFNβ protein was assessed at earlier time point (6 h) or in response to a lower dose of polyI∶C (0.1 µg/ml) (data not shown). We have also attempted to measure other members of type I IFNs as well. Namely, multi-subtypes of IFNα were measured by commercial ELISA [61]; however, levels of proteins detected in all supernatants were below the sensitivity of the ELISA and thus considered undetectable (data not shown).

### Enhanced polyI∶C-driven antiviral protection is associated with hyperactivation of IRF3 in Ad/Tr-cells

**Figure 4:**
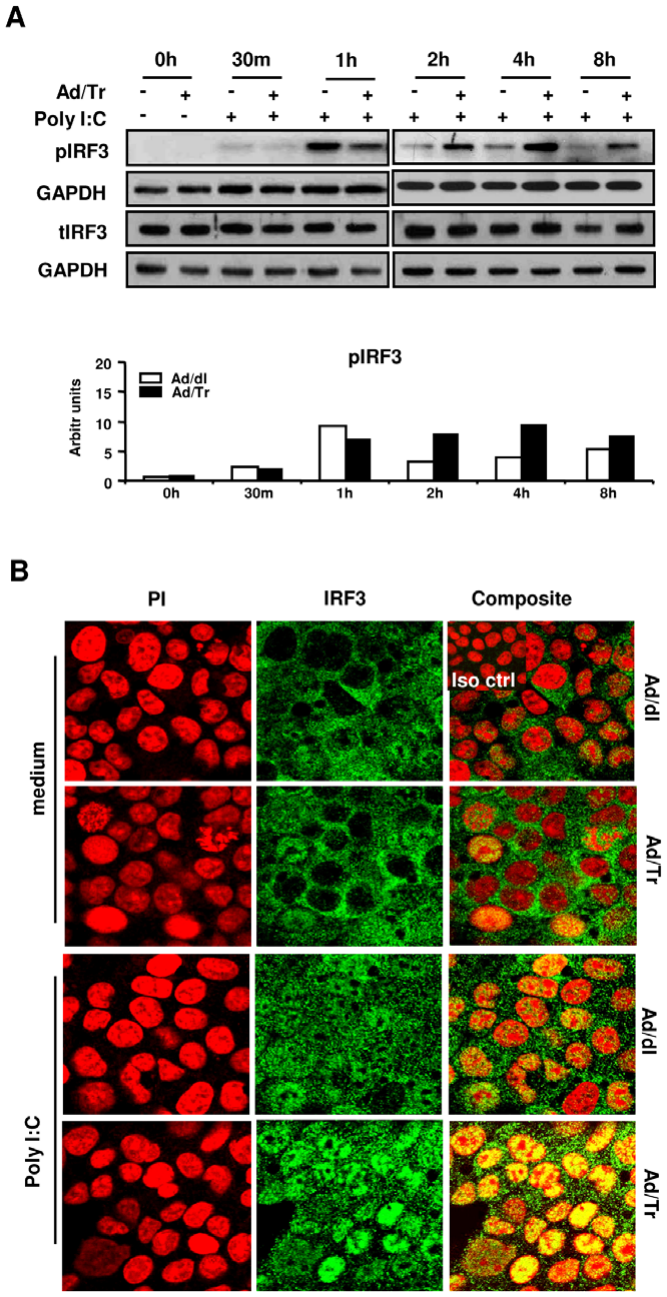
Enhanced polyI∶C-driven antiviral protection in Ad/Tr-cells is associated with hyperactivation of IRF3. (*A*) Western blots and their analyses of phosphorylated IRF3, total IRF3, and GAPDH proteins were performed from whole-cell extracts of HEC-Ad cells that were either left untreated or treated with 25 µg/ml polyI∶C during indicated time points. (*B*) Immunofluorescence analysis of IRF3 nuclear translocation following either medium alone or polyI∶C 25 µg/ml treatment for 4 h. Representative staining is shown for IRF3 (green), nuclear stain (PI) (red), and composite (yellow) at magnification 2520×. The data are representative of three independent experiments with similar results.

Phosphorylation and nuclear translocation of IRF3 are key events in the transcriptional activation of inducible ISG cellular genes [44], [48], and polyI∶C was previously shown to induce activation of IRF3 *in vitro* [39], [44]. Qualitative and quantitative analyses of phosphorylated IRF3 (pIRF3) showed that, compared to control cells, IRF3 phosphorylation in Ad/Tr-polyI∶C-treated cells was initially modestly reduced at 1 h, but then subsequently increased after 2 h (Figure 4A, pIRF3 WB panel and quantifying histogram) and remained increased for up to 24 h (data not shown). In contrast, no significant changes were evident in the total IRF3 (tIRF3) protein amount for Ad/Tr-cells, compared to controls (Figure 4A, tIRF3 WB panel). Confocal imaging at 4 h post polyI∶C stimulation (Figure 4B) corroborated WB findings of increased IRF3 phosphorylation in Ad/Tr-cells, since significantly more Ad/Tr-cells appeared with IRF3 translocated into the nucleus, compared to Ad/dl-cells (Figure 4B, polyI∶C panel). Taken together, these results suggest that exogenous expression of Tr/E promotes hyperactivation of IRF3 that is associated with increased antiviral protection of Ad/Tr-cells against VSV-GFP challenge.

### Tr/E significantly decrease mRNA expression of ISG56 in Ad/Tr-cells following polyI∶C stimulation

**Figure 5:**
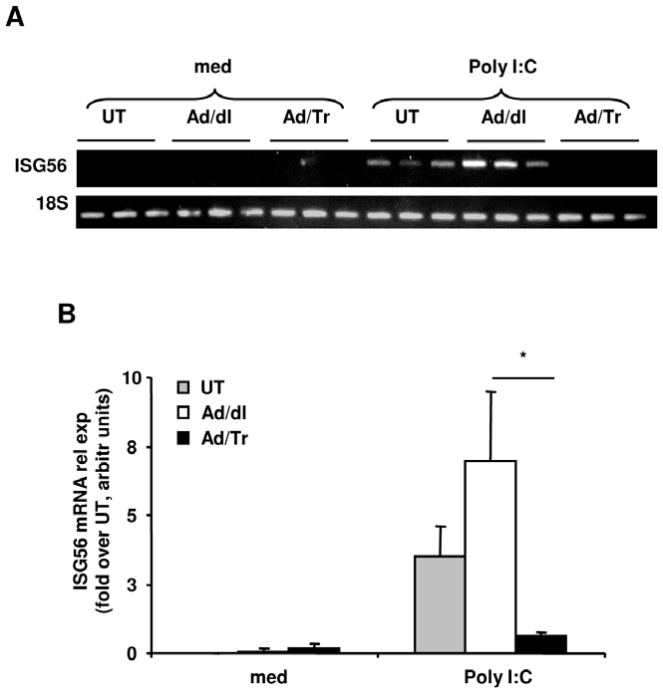
Exogenous expression of Tr/E significantly decreases mRNA expression of ISG56 in Ad/Tr-cells following polyI∶C stimulation. HEC-1A cells were either treated with medium alone or with MOI 50 of Ad/dl or Ad/Tr and incubated in presence of media or 25 µg/ml of polyI∶C. (*A*) At 6 h post treatment, total RNA was harvested and mRNA levels of ISG56 were assessed by conventional RT-PCR. (*B*) Quantification of ISG56 expression using ImageQuant software. Values are normalized to a housekeeping gene 18S in the same sample and presented as relative fold induction over untreated cells, shown in arbitrary units. The data are representative of at least two independent experiments performed in triplicate and are shown as the mean ± SD. Statistical analysis was performed using Student's *t* test with * representing significant difference between the groups, p<0.05.

ISG15 and ISG56 have been implicated in antiviral protection and linked to activation of IRF3 in response to polyI∶C [44], [62]. Results of RT-PCR demonstrated no difference in mRNA levels of ISG15 between Ad/Tr and Ad/dl groups after polyI∶C stimulation (data not shown). In contrast, mRNA expression of ISG56 (Figure 5A and 5B) appeared significantly reduced in Ad/Tr-cells in response to polyI∶C.

### Tr/E alter phosphorylation and transcriptional activity of NF-κB in Ad/Tr-cells in response to polyI∶C

**Figure 6:**
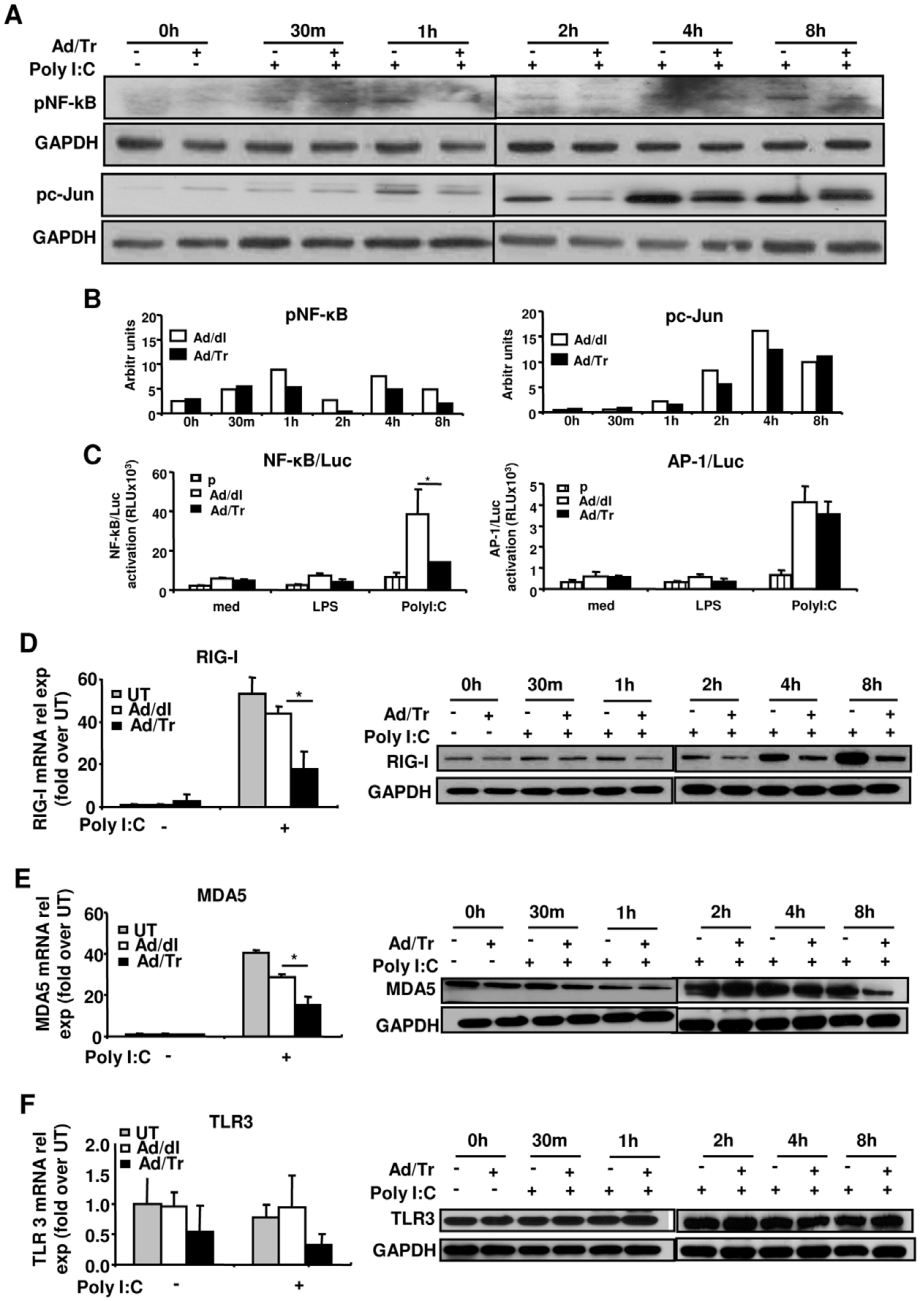
Altered NF-κB phosphorylation and transcriptional activity and reduced RIG-I and MDA5 expression in polyI∶C-treated Ad/Tr-cells. Western blots (*A*) and their quantification (*B*) of phosphorylated p65 subunit of NF-κB (pNF-κB) as well as c-Jun (pc-Jun) and GAPDH proteins were performed from whole-cell extracts of Ad-cells that were either left untreated or treated with 25 µg/ml polyI∶C during indicated time points. Transcription activity (*C*) of NF-κB (NF-κB/Luc panel) and (AP-1/Luc panel) was assessed by luciferase reporter assay by transfecting HEC-1A cells with pgkβ-Gal and pNF-κB-Luc or pAP-1-Luc plasmids (total DNA 40 ng per well) (p) alone or together with 50 MOI of Ad/dl or Ad/Tr overnight, washing, allowing to rest for 4 h and stimulating with 25 µg/ml polyI∶C and 1 µg/ml LPS for 4 h. Then luciferase and β-galactosidase activities were determined in cell lysates and expressed as relative luciferase units using galactosidase plasmid as normalization control. Data are presented as mean ± SD and are representative of three experiments for NF-κB and two - for AP-1. (*D–F, left panels*) Total RNA was harvested and mRNA expression of RIG-I, MDA5, and TLR3 was determined by real-time quantitative RT-PCR at 6 h post treatment with polyI∶C 25 µg/ml. Values are normalized to a housekeeping gene 18S in the same sample and presented as fold induction over untreated cells. (*D–F, right panels*) Western blot analysis of RIG-I, MDA5, TLR3, and GAPDH protein was performed from whole-cell extracts of Ad-cells that were either treated or not with 25 µg/ml polyI∶C during indicated time points. The data are representative of at least two independent experiments performed in triplicate and are shown as the mean ± SD. Statistical analysis was performed using Student's *t* test with * representing significant difference between the groups, p<0.05.

We next elucidated phosphorylation and activation of other transcription factors, including NF-κB and c-Jun, that have also been shown to contribute to antiviral responses [38], [41], [45]. Qualitative and quantitative results show that NF-κB p65 phosphorylation in Ad/Tr-cells was attenuated after 1 h post-polyI∶C exposure and remained decreased until 8 h, compared to the control group (Figure 6A, NF-κB WB panel and Figure 6B, NF-κB quantifying histogram). Our results further revealed that overall c-Jun phosphorylation was only transiently reduced in Ad/Tr-cells between 1 h and 4 h and returned to levels comparable to such of controls at 8 h post polyI∶C treatment (Figure 6A, pc-Jun WB panel and Figure 6B, pc-Jun quantifying histogram). These results prompted us to next evaluate the effect of Tr expression on transcriptional activity of NF-κB and c-Jun, which was assessed by luciferase reporter gene assay. Data shown in Figure 6C, NF-κB/Luc panel, demonstrate that transcriptional activity of NF-κB in Ad/dl-polyI∶C-treated cells was significantly induced at 4 h, compared to untreated and LPS-treated Ad/dl-cells. However, polyI∶C-induced NF-κB transcriptional activity in Ad/Tr-cells was significantly reduced, compared to Ad/dl-cells. In contrast, transcriptional activity of AP-1 was not different between the groups following stimulation with either of the ligands (Figure 6C, AP-1/Luc panel). Collectively, our data show that Tr/E expression attenuated NF-κB activation not only at the phosphorylation level, but also at the level of its transcriptional activity, which was more pronounced compared to changes observed for c-Jun/AP-1.

### Tr/E significantly reduce levels of dsRNA sensors RIG-I and MDA5 in polyI∶C-treated Ad/Tr-cells

PolyI∶C-induced antiviral protection of primary genital ECs was previously associated with heightened expression of TLR3 [40]. Thus, we evaluated the expression levels of dsRNA sensors in Ad/Tr-cells following polyI∶C treatment. Figures 6D–F show that in contrast to TLR3, polyI∶C stimulation induced a significant (over 40 times) increase in mRNA expression of RIG-I and MDA5 (Figure 6D–E, left panels) in all UT and Ad-cells. Further, compared to UT and Ad/dl-cells, Ad/Tr-cells surprisingly had significantly attenuated expression of RIG-I and MDA5 at both mRNA and protein levels (Figure 6D–E, left and right panels). RIG-I expression in Ad/Tr-cells appeared to be affected at earlier time point, around 1 h, compared to MDA5 expression that was attenuated at 8 h after polyI∶C stimulation. Moreover, reduced expression levels of RIG-I and MDA5 were sustained for up to 24 h following polyI∶C treatment (data not shown). The expression of TLR3, however, was not different between the groups (Figure 6F). Collectively, these data indicate that mRNA and protein expression of RIG-I and MDA5 are increased in response to polyI∶C stimulation, but the magnitude of expression is reduced in Ad/Tr-cells. Further, that we observed differential pattern of expression among RIG-I, MDA5, and TLR3 could indicate either a different kinetics of responses of these sensors, or that each RIG-I, MDA5, and TLR3 respond differentially to polyI∶C, known to be a mixture of various lengths of dsRNA [43].

### IRF3 is required for polyI∶C-driven antiviral protection in Ad/Tr-cells

**Figure 7:**
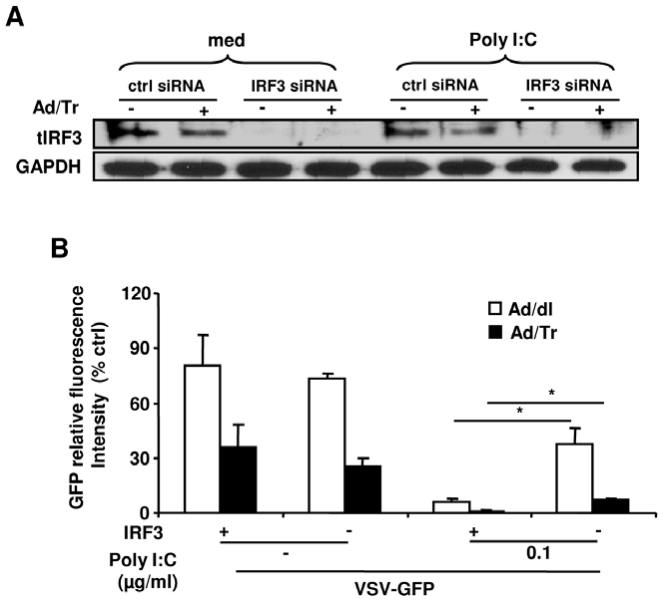
IRF3 is required for polyI∶C-driven antiviral protection in Ad/Tr-cells. (*A*) HEC-1A cells were left untreated or transfected with a non-targeting control siRNA (ctrl siRNA), or IRF3 siRNA (IRF3 siRNA) for 48 h. Two days after siRNA delivery, HEC-1A received MOI 50 of Ad/dl or Ad/Tr followed by 25 µg/ml of polyI∶C treatment for 24 h and Western blot analyses of total IRF3 (tIRF3) and GAPDH proteins were performed from whole-cell extracts 96 h post-transfection. (*B*) Twenty four hours following polyI∶C treatment, cells were infected with MOI 1 of VSV-GFP for another 24 h. Levels of GFP fluorescence were visualized and quantified using a Typhoon scanner. The fluorescence reading of treated cultures was normalized to untreated (control) cultures and presented as percentage relative fluorescence. The data are representative of two independent experiments performed in triplicate and are shown as the mean ± SD. Statistical analysis was performed using Student's *t* test with * representing significant difference between the groups, p<0.05.

We next determined whether IRF3 was required for enhanced polyI∶C-driven antiviral state in Ad/Tr-cells. IRF3 was knocked down by utilizing IRF3-specific small interfering RNA and cells were subsequently treated, or not, with polyI∶C before challenging with VSV-GFP. The greatest knockdown efficiency was observed between 72 h and 96 h post transfection (data not shown). Results of WB demonstrated no apparent expression of tIRF3 after siRNA treatment (Figure 7A). Further, VSV-GFP replication in polyI∶C-untreated Ad/dl- and Ad/Tr-cells was not significantly altered when IRF3 was knocked down (Figure 7B). This observation suggests that IRF3 is dispensable for antiviral defense against VSV-GFP infection in both Ad/dl and Ad/Tr groups in the absence of polyI∶C treatment and that markedly reduced viral replication in Ad/Tr-cells was attributed to other, yet unidentified, factor(s). In contrast, when polyI∶C was added, VSV-GFP replication was initially markedly reduced in both Ad/dl and Ad/Tr groups in the presence of IRF3, but then significantly increased in the absence of IRF3; yet, Ad/Tr-cells still remained more protected than Ad/dl group. This observation implies that enhancement of polyI∶C-triggered antiviral protection in Ad/Tr-cells was only partially dependent on IRF3. Figure 7B also showed that VSV-GFP replication was restored about 50% of the original viral load detected in each of the groups in absence of IRF3 and polyI∶C stimulation, suggesting that additional mechanisms/factors were contributing to polyI∶C-induced antiviral protection. Collectively, these findings indicate that IRF3 plays an equally important role in antiviral protection in both Ad/dl and Ad/Tr groups, and that the presence of IRF3 is important, but not essential, for enhanced polyI∶C-induced antiviral protection in Ad/Tr-cell, as other factors, perhaps upstream of IRF3, may also be contributing to this protection.

### Tr/E significantly reduce pro-inflammatory cytokines in ECs following polyI∶C and LPS stimulation

**Figure 8:**
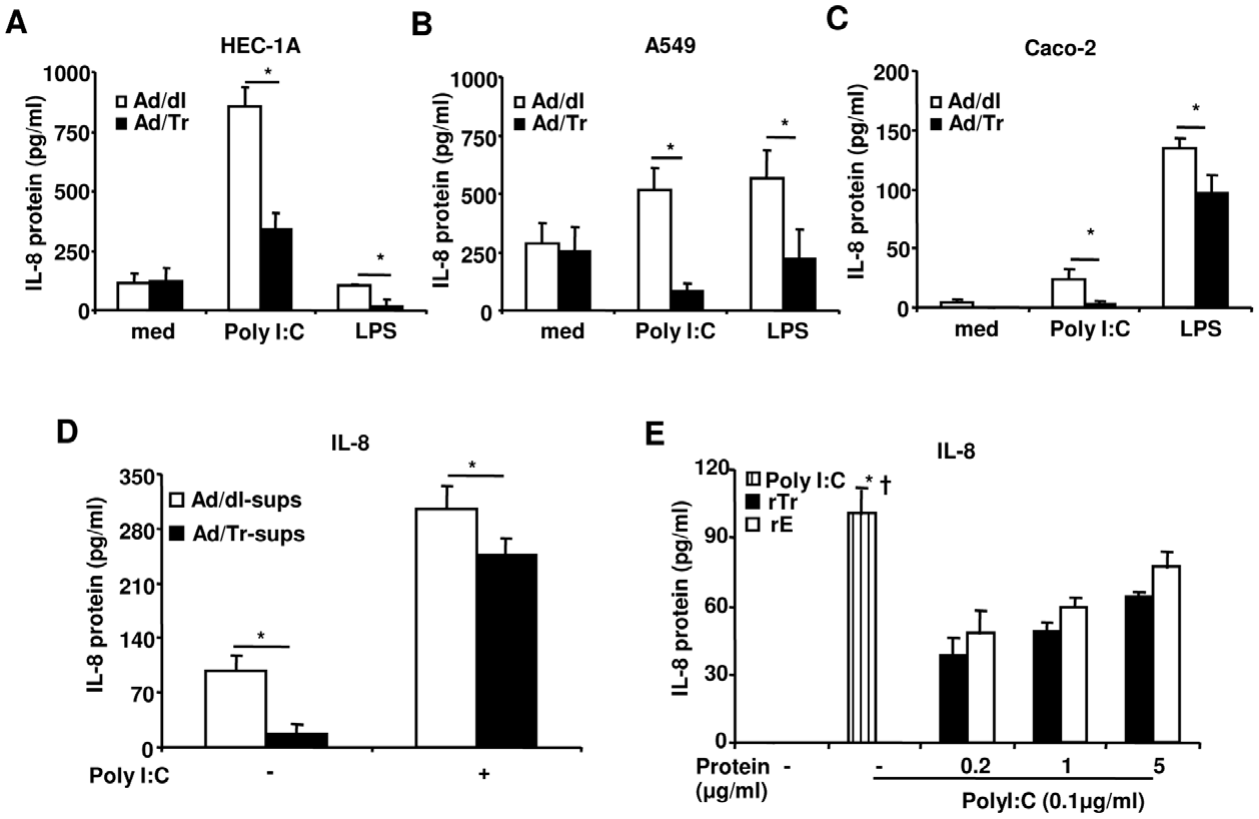
Tr/E significantly reduce protein secretion of IL-8 in ECs following polyI∶C and LPS stimulation. Secretion of IL-8 in supernatants from HEC-1A (*A*), A549 (*B*), and Caco-2 (*C*) cells initially infected with MOI 50 of Ad/dl or Ad/Tr and subsequently stimulated with either 25 µg/ml of polyI∶C or 1 µg/ml of LPS for 24 h. Following stimulation, supernatants were tested for IL-8 secretion by ELISA. Data are representative of at least two independent experiments performed in triplicate and expressed as the mean ± SD, shown in pg/ml. Statistical analysis was performed using Student's *t* test with * representing significant difference between the groups, p<0.05. (*D*) Secretion of IL-8 in supernatants from HEC-1A cells that were pre-treated with HEC-Ad/dl and HEC-Ad/Tr supernatants before polyI∶C stimulation. HEC-1A received 50 µl of concentrated supernatants containing around 10 µg/ml of Tr/E in Ad/Tr supernatants and 0.004 µg/ml of Tr/E in Ad/dl sups for 1 h, to which additional 50 µl of medium alone or polyI∶C to the final concentration of 0.1 µg/ml were added for 24 h. (*E*) Secretion of IL-8 in supernatants from HEC-1A cells treated with commercial 6×His-Tr (rTr) or in-house HAT-E (rE) for 1 h and then stimulated with medium alone or 0.1 µg/ml of polyI∶C for 24 h. A lower dose of polyI∶C was used in (*D*) and (*E*), since anti-inflammatory effects of secreted/soluble proteins, compared to Ad/Tr-cells, were less potent. Statistical analysis was performed using ANOVA, and * representing significant difference between the polyI∶C group and rTr groups; † representing significant difference between the polyI∶C and rE groups, p<0.05.

PolyI∶C stimulation induces not only antiviral, but also pro-inflammatory factors [39], [40] that are regulated by viral sensors and transcription factors, including NF-κB and c-Jun. We therefore assessed levels of pro-inflammatory mediators IL-8, TNFα, and IL-6 in Ad/Tr-cells treated with polyI∶C. Figures 8A–C demonstrate that Ad/Tr-cells, regardless of their origin (i.e., genital HEC-1A or gut Caco-2, etc.), secreted significantly lower levels of IL-8 24 h after polyI∶C and LPS treatment, compared to controls; TNFα and IL-6 were similarly reduced in Ad-cells in response to polyI∶C (data not shown). Interestingly, stimulation of HEC-1A with LPS did not produce a significant increase in IL-8, compared to untreated cells, possibly due to a low baseline expression of TLR4 in the genital EC [39]. These results clearly indicate that Tr/E controlled the release of pro-inflammatory mediators in ligand-treated cells. Interestingly, Tr/E secreted by Ad/Tr-cells appeared to significantly contribute to reduced IL-8 levels. Indeed, Tr/E neutralization with specific anti-Tr/E TRAB20 (HyCult Biotech) antibodies (under the control of antiprotease assay) that were added to Ad/Tr-cells 1 h prior to 0.1 µg/ml of polyI∶C treatment and subsequently co-cultured with polyI∶C for additional 24 h to neutralize any secreted Tr/E, led to significantly higher polyI∶C-induced IL-8 secretion (up to 40%, p = 0.03) in Ad/Tr-cells (data not shown). Figures 8D and 8E further show that HEC-1A cells pre-treated with either Ad/Tr-sups, rTr or rE before subsequent co-culture with polyI∶C also released lower levels of IL-8 in response to polyI∶C. Of note, anti-inflammatory effects of secreted/soluble proteins, compared to Ad/Tr-cells, appeared to be less potent and context-dependent, which forced us to use a lower dose of 0.1 µg/ml of polyI∶C. Additionally, either Ad/Tr-sups or rTr/rE alone did not trigger any significant IL-8 production in absence of polyI∶C. Collectively, our findings indicate that Tr/E are capable of inhibiting inflammatory responses in ECs from various sources and against both viral and bacterial PAMPs, and that secreted/soluble Tr/E significantly contribute to reduction in polyI∶C-induced IL-8 secretion. However, stimulation of immune responses can also be observed depending on experimental conditions.

## Discussion

WAP proteins, including antiproteases Tr/E, SLPI, and ps20, are pleiotropic molecules known to play multiple and significant roles in health and disease [8], [10], [11]. Indeed, ps20 has been reported as a potential diagnostic marker in prostate cancer [63] and as a novel negative signature protein in HIV infection [64]. In contrast, SLPI and Tr/E show significant therapeutic potential in atherosclerosis as well as cardiovascular [25], [65], [66], [67], lung [68], [69] and gut disorders [66], [70]. Additionally, higher levels of Tr/E in CVLs of HIV-resistant CSWs [29] and the testing of the Lactobacilli-based elafin delivery system for combating STIs in the FGT [71] would further support this notion. Our data showed that delivery of Ad/Tr to HEC-1A cells resulted in secretion of functional Tr, while both Tr/E were detected following treatment of these cells with polyI∶C. Moreover, polyI∶C treatment further resulted in Tr/E-enhanced antiviral protection and significantly reduced pro-inflammatory IL-8, IL-6, TNFα that were associated with lower expression of viral innate sensors RIG-I and MDA5 and altered NF-κB activation in Ad/Tr-cells. Notably, increased antiviral protection was due in part to Tr/E ability to act directly on virus or by modulating polyI∶C-driven cellular antiviral responses. Interestingly, such Tr/E-augmented cellular responses triggered by polyI∶C were partially mediated through IRF3 activation, but not higher induction of IFNβ, thus suggesting multiple antiviral mechanisms of Tr/E and the involvement of alternative and still unidentified factors or pathways.

This is the first study that comparatively assessed the presence and potential mechanisms of antiviral activity of each Tr/E. Here, we presented evidence showing two distinct, but likely complimentary antiviral properties of Tr/E: (i) direct antiviral activity exerted during the virus/cell interaction and targeting virus, but not cells; and (ii) indirect and cell-associated immunomodulatory activity, targeting polyI∶C-triggered cellular antiviral responses. The virus-mediated activity was observed in the absence of polyI∶C stimulation and in the presence of Tr in Ad/Tr-cells (presumably present in both Ad/Tr-sups and Ad/Tr-cells). Because only Tr was detected in polyI∶C-untreated Ad/Tr-cells, and because the expression of IFNβ was low and not different between untreated Ad/dl and Ad/Tr groups, we conclude that in the absence of polyI∶C, Tr alone was mediating antiviral activity in Ad/Tr-cells by acting either directly on virus or indirectly through cells. However, we failed to transfer this direct antiviral effect of Tr via Ad/Tr-sups, possibly due to the absence of an additive protective effect from augmented intracellular Tr (as would be expected in Ad/Tr-cells),or due to an inefficient delivery of Tr in supernatants and a “diluting" effect from other antiviral factors released in response to Ad/dl delivery (Fig. 2E, insert).

Additionally, the presence of both Tr/E, as in rTr preparation, was also protective against VSV-GFP challenge in the absence of polyI∶C treatment. Although no reports describing direct antiviral activity of Tr are published to date and no precise mechanisms of Tr/E direct antiviral effect have been identified, our observation with rTr is in line with Ghosh *et al.* findings, describing a direct antiviral effect of E as a mode of action against HIV [6]. Interestingly, rTr also appeared to have only virus- and not cell-mediated protective effects, similar to E mentioned earlier [6]. In contrast, a close WAP member SLPI, was shown to have only cell-mediated antiviral effects, at least against HIV [72] and herpes simplex virus (HSV) [73]. Examples of same-family members having differential antiviral mechanisms have also been described for other innate molecules, such as human defensins against HIV [74], [75] and HSV [76]. These reports suggest that molecules even from the same group, may possess their own, potentially different in potency and targets, exquisite antiviral activities and yet still uniquely contribute to overall mucosal protection against STIs.

Our results further suggest that the presence of both Tr/E might be required for each virus- (direct) and cell-associated (indirect) antiviral effect, as was evident from our data using Ad/Tr-cells, Ad/Tr-sups, and rTr, indicating that perhaps the most efficient antiviral protection depends on collaborative work of both Tr/E. Interestingly, when HIV-susceptible, but uninfected, CSWs were followed prospectively, those who remained HIV-negative had elevated levels of both Tr/E detected in CVLs [29]. Additionally, when characterizing the specificity of proteins secreted after Ad/Tr infection, our ELISA and WB results also showed that Ad/Tr-cells secreted both Tr/E independently in response to polyI∶C, while only Tr was detected without polyI∶C stimulation. Although Ghosh *et al.* also reported that primary uterine EC produced Tr/E in response to polyI∶C [6], the independent production of E was never demonstrated. Further, only Tr (13–16 kDa) was previously identified in supernatants from LPS-stimulated alveolar ECs [37]. On conjuncture, these results suggest that expression of each Tr/E could be a tissue/cell- or ligand-specific defense mechanism against an unknown protease that was potentially activated in response to a viral ligand. It might be important in the future to clarify whether primary genital EC from the FGT produce each Tr/E independently in response to polyI∶C, similarly to Ad/Tr-cells.

It is unclear why tested rE failed to show antiviral activity against VSV-GFP; it could be attributed, however, to a HAT-tag insertion at the N-terminus of rE. Indeed, all rTr, rE, and secreted Tr were equally functional against HNE (data not shown) and capable of inhibiting IL-8 production in response to polyI∶C (Figure 8). Yet, while rTr with a His-C-terminus tag exhibited antiviral activity, rE with a HAT-N-terminus tag did not. This observation suggests that blocking N-terminus, but not C-terminus, appears to be critical for antiviral activity of rE. An earlier study by McMichael *et al.* supports this argument, since they showed that the N-terminus of Tr had a better affinity for LPS than its C-terminus end [28]. Additionally, it is unclear why we observed increased levels of IL-8 with higher concentrations of rTr and rE. But one possible explanation could be that the proteins were initially delivered and left on cells in serum-free conditions, thus promoting the activation of pro-inflammatory events as was previously shown for Tr/E in response to LPS [28].

The second antiviral property of Tr/E observed in our study was an indirect cell-mediated immunomodulatory activity of Tr/E, targeting polyI∶C-induced antiviral cellular responses. In contrast to responses to bacterial or pro-inflammatory stimuli [25], [26], the scope and specific mechanism(s) of viral ligand-triggered immunomodulatory activity of Tr/E have never been fully investigated. Our data demonstrate that this indirect cell-associated activity is targeting viral recognition through modulation of RNA helicase expression as well as the induction of key inflammatory and antiviral innate signaling pathways and mediators.

Our results showed that polyI∶C-triggered antiviral cellular protection was significantly enhanced in the presence of Tr (in Ad/Tr-sups) and Tr/E (in Ad/Tr-cells and in rTr). We also showed that polyI∶C-mediated activation of IRF3 was further induced in Ad/Tr-cells, compared to controls, whereas IFNβ expression was dampened. Interestingly, human β defensin 3 [77] and cathelicidin LL37 [78] that were previously shown to have antiviral, including anti-HIV, activity [79], [80], were also reported to inhibit IFNβ production *in vitro* in response to LPS and polyI∶C, respectively. These observations further support our results and strengthen the earlier argument of Tr/E acting either directly against VSV-GFP or through cells and additional factors/pathways in Ad/Tr-cells. Furthermore, moderation of immune-inflammatory responses and thus curbing undesirable immune activation might be one of the protective mechanisms of innate antimicrobials at mucosal sites. Additionally, in searching for ISGs typically associated with antiviral protection and IRF3 activation [62], we found that expression of ISG15 was not significantly changed, unlike ISG56 being reduced and in agreement with IFNβ data. It is not entirely understood why such discordance was observed; however, it could be due to the fact that ISG15 was shown to be regulated by either IRF3 or IFNβ [44], [81], unlike ISG56 that was shown to be under the regulation of IFNβ or viruses [82], [83] and thus following IFNβ pattern of induction as shown in our study. The alternative explanation could be that these two genes follow a different temporal pattern of activation that was overlooked here.

This is the first report on the involvement of serine antiproteases, Tr/E in particular, in antiviral signaling pathways. As no prior data are available on the role of Tr/E in IFNβ and IRF3 induction, further and more detailed investigations might be required to explain why in Ad/Tr-cells IFNβ and ISG56 levels were reduced while IRF3 activation was increased. We hypothesize, however, that this phenomenon could be an attempt of Tr/E to control antiviral inflammatory events through RIG-I/MDA5 and NF-κB downregulation while increasing cellular protection through activation of IRF3 and/or alternative factors or pathways. Although most of the studies show ISG56 to be associated with upregulated IRF3 [62], our finding is in line with data from Li *et al.* showing that a knockdown of ISG56 was associated with increased IRF3 activation and inhibition of VSV-GFP replication [84] as a result of ISG56 mediating MITA-TBK1 interaction and subsequent downstream activation of IRF3. It is also possible that alternative factors/pathways, in addition to IRF3, regulate IFNβ and ISG56 expression and contribute to Tr/E-enhanced antiviral protection, which is also supported by our IRF3 siRNA data. Collectively, these data indicate that in the presence of Tr/E, antiviral protection is increased and that direct or indirect antiviral effect(s) of Tr/E depend, but not exclusively, on IRF3 and other factors, perhaps upstream of IRF3.

Inflammation is one of the leading factors predisposing to acquisition and disease progression of STIs in the FGT [52], [53], [85]. This notion is supported by the fact that “immune quiescence" and reduced immune activation are crucial for resistance against STIs [86], while dysregulated TLR expression and immune-inflammatory responses are detrimental [52], [53], [85]. Here, we showed that Tr/E individually or as a mixture, as well as in Ad/Tr-cells and as secreted/soluble proteins in Ad/Tr-sups, were capable of reducing IL-8, IL-6, and TNFα expression in response to polyI∶C. Moreover, in Ad/Tr-cells we also observed significantly reduced activation and transcriptional activity of NF-κB. The IL-8 inhibitory effect was not specific to human endometrial ECs, or to polyI∶C, indicating that similar effects could be observed at other mucosal surfaces and in response to different microbial ligands. We further showed that, compared to controls, mRNA and protein levels of RIG-I and MDA5 (mainly at a later time point), but not TLR3, were significantly diminished in response to polyI∶C and in presence of Tr/E. Immunomodulatory properties of both Tr/E demonstrated in models of pro-inflammatory and bacterial (LPS) stimulations were shown to depend on inhibition of NF-κB and AP-1 activation [25], [26], thus further supporting our NF-κB data. However, Tr/E inhibitory effect targeting antiviral immune responses, including viral sensing, has not been previously reported. Hence, modulation of expression of RIG-I, MDA5, and pro-inflammatory mediators shown here could represent novel antiviral functions of Tr/E, possibly even executed at different levels, namely receptors and transcription factors. That we observed differential pattern of RIG-I, MDA5, and TLR3 expression could indicate either different temporal kinetics of responses of these sensors, or that each RIG-I, MDA5, and TLR3 respond differentially to polyI∶C, being a mixture of variable lengths of dsRNA [43]. Further, the lack of polyI∶C-triggered TLR3 induction in HEC-1A compared to primary genital ECs, also likely reflects tissue or structure-dependent differences between the cells, suggesting that primary genital ECs may exhibit distinct results. Collectively, these observations suggest that Tr/E can alter innate viral recognition and mounting of antiviral immune-inflammatory responses.

The precise mechanism(s) of immunomodulatory effects of Tr/E, as both secreted and/or intracellularly expressed proteins, on viral sensors, cytokines, and IFNβ in response to polyI∶C treatment is still largely unknown. This is partly because the existence of the cognate receptor for Tr/E remains elusive, and it is equally unknown whether Tr/E require a receptor to function. We propose that reduced levels of IL-8, IL-6, TNFα and IFNβ in Ad/Tr cells in response to polyI∶C are likely a result of overall attenuation of RIG-I and MDA5 levels, as they are known to regulate the expression of pro-inflammatory and antiviral mediators [4], [41], [87] through activation of main signaling pathways, such as NF-κB that is downregulated in our study [41]. It remains to be elucidated, however, how Tr/E specifically inhibit RIG-I and MDA5 expression. It is plausible that Tr/E directly bind to polyI∶C, as was shown for binding of LL37 to polyI∶C [78], as well as Tr/E binding to LPS [28]. Such an interaction may alter binding/recognition of polyI∶C by its cognate receptors, including cell-surface scavenger receptor A or intracellular sensors RIG-I, MDA5, and TLR3, which in turn could explain our reduced expression levels of RIG-I and MDA5. Another possible site of inhibition by Tr/E could be downstream of receptors/viral sensors and involve Tr/E binding to DNA and competing for specific DNA binding sites with transcription factors including NF-κB, as was shown for SLPI as one of its anti-inflammatory mechanisms in response to LPS [88].

A noteworthy observation of this study is that while Tr from Ad/Tr-sups and rE were found functional against HNE and able to inhibit polyI∶C-induced IL-8 production, they did not show any antiviral activity, suggesting that antiprotease, anti-inflammatory, and antiviral activities of the tested proteins may not necessarily be co-dependent or predictive of each other; nonetheless, they can be complimentary. This observation is supported by earlier reports, showing both a protease non-inhibitory N-terminus and an inhibitory C-terminus of Tr exhibiting comparable antibacterial and antifungal functions [22], [24]. In contrast, Mulligan *et al.* showed that SLPI Gly(72) mutant, unlike other mutants tested in that study, lost its *in vivo* immunosuppressive activity against NF-κB activation and neutrophil recruitment in the lungs that appeared to be most closely related to SLPI's trypsin-inhibiting activity [89]. Although being an important property of both Tr/E, the inhibition of HNE activity is not considered a critical function for our studies, since epithelial cells do not make neutrophil elastase [90] and thus, the earlier discussed Tr/E-mediated changes are most unlikely attributed to antielastase activity of the proteins. The above observations indicate that perhaps additional structure-function studies might be warranted in the future to specifically address the cross-talk between antiprotease, anti-inflammatory, and antiviral properties of Tr/E and their specific roles in defense against viruses.

Overall, our data support and further extend earlier observations on immunomodulatory effects of Tr/E [25], [26]. This work demonstrates that in genital ECs and in response to polyI∶C, Tr/E antiviral effects are mediated through direct or virus targeting activity and indirect or cell-associated immunomodulatory function(s) that target host innate recognition and mounting of antiviral and inflammatory responses. While dampening of IFNβ, a key antiviral mediator, may seem counterintuitive and detrimental to antiviral defenses, our findings suggest that directly or indirectly increased antiviral protection and moderated, or finely-tuned, inflammation, might be more advantageous to a host in the context of viral exposure. In conclusion, this study clearly demonstrates the importance of Tr/E in antiviral protection. Our findings also propose the existence of multiple targets and potentially several and unique modes of action for each of the proteins, which warrant additional research in the future.
